# Alkali-activated slag concrete with paper industry waste

**DOI:** 10.1177/0734242X20983890

**Published:** 2021-02-03

**Authors:** Maria Mavroulidou, Shamil Shah

**Affiliations:** 1 London South Bank University, UK; 2 Highway Design & Maintenance, Environment & Infrastructure, Hertfordshire County Council, UK

**Keywords:** Solid waste management, ground granulated blast furnace slag, paper sludge ash, alkali-activated cements, concrete

## Abstract

Pulp and paper manufacturing and recycling industries are a resource-intensive sector, generating 25–40% of the annual municipal solid waste worldwide. Waste includes abundant volumes of paper sludge, as well as the product of its incineration, namely paper sludge ash. These two waste materials are both predominantly landfilled. There is thus a drive for additional valorisation routes for these materials. This short communication focuses on the potential use of paper sludge ash in alkali-activated cement concrete; this type of concrete was estimated to potentially reduce CO_2_ emissions by up to 5–6 times, while it can also incorporate waste materials or industrial by-products in its composition. The paper presents a laboratory study assessing the feasibility of structural alkali-activated cement concrete with ground granulated blastfurnace slag (a by-product of steel production) and paper sludge ash. Paper sludge ash is used mainly as a source of Ca(OH)_2_ in the alkaline activator solution, and secondly as an additional source of aluminosilicates. A number of factors potentially affecting the activation process and the resulting concrete quality were investigated, including different dosage of activators, curing conditions and curing time. Mixes with paper sludge ash in the activator system developed high early concrete strengths at ambient temperatures and maintained adequate strengths for structural concrete. Further mix optimisation and mechanical and durability testing, accompanied by material characterisation, are required to establish the advantages of using this waste material in structural alkali-activated cement concrete.

## Introduction

Pulp and paper manufacturing and recycling industries are a resource-intensive sector, generating 25–40% of the annual municipal solid waste worldwide. In Europe, pulp production reached 36.5 million tonnes and the paper used for recycling 47.6 million tonnes (Paperchain, 2018). Although in the last decade the sector has made an intensive effort to reduce landfilling and waste disposal (mainly by fibre recycling and energy recovery from different waste streams), there are insufficient recycling or recovery routes for the waste, including the ashes generated in the different energy recovery systems. It is thus stored on-site and/or landfilled, incurring high costs to the companies (e.g. storage costs between €15 tonne^-1^ and €70 tonne^-1^ for non-hazardous solid waste were reported in a number of EU countries; Paperchain, 2018). Due to the high concentration of the sector, local markets cannot absorb the large amounts of waste produced at one single point. The construction and building materials sector, consuming 5.4 billion tonnes of raw materials yearly (Paperchain, 2018), and in particular concrete, the most widely used material in construction after water, could provide an ideal valorisation opportunity for such waste. This short communication investigates the feasibility of incorporating paper sludge ash (PSA) from the incineration of paper mill sludge (the main waste stream of the deinking and re-pulping of paper) in alkali-activated cement (AAC) systems for structural concrete. AAC systems are binder systems produced by the reaction of an alkali metal source with a solid (alumino-)silicate; alkali metal ions raise the pH of the mix, accelerating the solid precursor dissolution. AAC have gained increased interest as more economical and environmentally sustainable alternatives to Portland cement, potentially reducing CO_2_ emissions by up to 5–6 times (Davidovits, 2013) and valorising waste materials in their production. PSA is mainly a calcium aluminosilicate. It has cementitious properties and a pH = 12.3–12.4 due to its high free CaO content (typically 10%, Tagnit-Hamou et al., 2015; but free CaO > 20% was reported in Doudart de la Grée, 2012). In Europe, it is thus classified as hazardous waste as it is corrosive (EU Directive 2008/98/EC Annex III, criterion H8). However, in terms of hazardous substance leaching, it would have generally been classified as inert waste according to the solid waste disposal criteria of 2003/33/EC Decision (Dunster, 2007; European Commission [EC], 2003) and is plastic-free. It is produced in controlled heat and power (CHP) plants according to the EU Waste Incineration Directive (EC, 2000), so that dioxins and furans are kept to trace levels. Incineration is done mainly to reduce the volume of paper sludge waste (80–90% reduction) which is predominantly landfilled, although some alternative options are also possible (e.g. land-spreading of sludge as an agricultural fertiliser and use as fuel (Class 2 – alternative liquid fuel)) (Mavroulidou et al., 2019a). The increasing amount of PSA going to landfill (e.g. in the UK 4 out of 40 paper mills alone generate 140 ktonnes of PSA annually, which is mostly landfilled; Spathi, 2015) has caused environmental concerns and high costs to industry, thus the need for more sustainable alternative management options. In terms of waste management, an advantage of incorporating PSA in AAC, suitable for precast concrete, is that it circumvents the need to find construction projects located next to the production site to reduce transport costs, avoiding modifications of site equipment (e.g. to mix PSA with water) or dust generation (ash particles) potentially causing harm to plants or crops next to construction sites. In the PSA used in this study, the principal oxides are *c*. 60% lime (CaO), *c*. 25.7–16.43% silica (SiO_2_), *c*. 18.86–9.05% alumina (Al_2_O_3_); Fe_2_O_3_ is *c*. 0.9–0.41% (Mavroulidou, 2018). The combined content of SiO_2_, Al_2_O_3_ and Fe_2_O_3_ is <50%; therefore, PSA is not a pozzolan. Attempts to activate this PSA using NaOH did not lead to significant strength gains (Martynková and Mavroulidou, 2015), consistently with Bernal et al. (2014), who did not observe the formation of geopolymer under the activation conditions they used (for the same PSA). Therefore in this study, the PSA will be considered mainly as an alkaline activator of ground granulated blastfurnace slag (GGBS) (a by-product of steel production). The use of this PSA as an activator of GGBS for soil stabilisation cements is very promising (Mavroulidou et al., 2019b, 2020). Ca(OH)_2_-activated GGBS AAC concrete was also produced successfully (e.g. Yang et al., 2012). The concept of using PSA in concrete is therefore well-founded and some previous publications (e.g. Mozaffari et al., 2009) used it as a GGBS activator for low strength concrete. However, for structural concrete PSA was mainly proposed as supplementary cementitious material replacing Portland cement in modest amounts (e.g. Fauzi et al., 2016; Fava et al., 2011; Kadu and Gajghate, 2016; Mavroulidou and Awoliyi, 2018; Mavroulidou et al., 2013; Rajgor and Pitroda, 2013). Its use in AAC formulations for structural concrete is mostly unexplored with very rare exceptions (e.g. Pachamuthu and Thangaraju, 2017, who used as precursors mixes with pulverised fuel ash and PSA). It is thus the specific innovative aspect of this study.

## Materials and methods

PSA was provided by a newspaper recycling company in the south-east of England from the incineration of non-hazardous paper sludge from the secondary processing stage of recycled fibres (cleaning with 85°C water and bleaching to remove any ink left). The mixes of alkaline activators contained (a) analytical grade potassium hydroxide solution (KOH) and waterglass (WG) (i.e. a sodium silicate (Na_2_SiO_3_) solution of a modulus M = SiO_2_/NaO_2_ = 2); (b) KOH and WG plus PSA, partially replacing KOH; (c) WG only, to isolate its effect from that of KOH and PSA; and (d) WG plus PSA. PSA used on its own to activate the GGBS was not considered as previous studies showed lower strengths than those required for structural concrete (e.g. Mavroulidou et al., 2013; Mozaffari et al., 2009). GGBS was provided by Hanson Regen; its suitability for AAC and the chemical composition of all materials were discussed in Mavroulidou and Martynková (2018). Aggregates used were river sand (5 mm maximum size) and gravel (10 mm maximum size). Table 1 shows the concrete mix designs. KOH and KOH+WG activators were prepared one day before casting. PSA slurry (or powder) was mixed with the rest of the ingredients on the day of concrete preparation. All dry ingredients were mixed in a concrete mixer for 2 min; half of the water was then added to the mix followed by the alkaline solution/PSA slurry; the rest of the water was then added and mixing continued for 4 min. The slump of fresh concrete was measured (Table 1) immediately after mixing (British Standards Institution [BSI], 2009a). Mixes with PSA were generally stiff to very stiff (slumps of 30 mm or less), the same as when PSA is used as a supplementary cementitious material in Portland cement (e.g. Yousuf et al., 2014; Ahmad et al., 2013; Mavroulidou and Awoliyi, 2018) due to the high water demand of PSA caused by its high porosity and free lime content (Doudart de la Grée et al., 2018). Although initially fluid, mixes with WG became unworkable and difficult to place in moulds within 15 minutes from mixing (consistently with Puertas et al., 2014) due to early formation of C-S-H gel. However, mix 4 was remixed for another two minutes, which increased the slump to 195 mm. Longer mixing will thus be adopted in the procedure in future studies. The fresh concrete samples were cast in moulds and compacted in three layers, using a vibrating table. Four different curing methods were studied: (a) Method 1 (Adam, 2009): curing in moulds at room temperature for 24 h, demoulding and water-curing for six days at 20°C, then constant humidity curing at room temperature; (b) Method 2: constant humidity curing (samples covered by an impermeable membrane); (c) Method 3: curing at 65°C for 5.5 hours (Brough and Atkinson, 2002), then (after cooling overnight) water-curing at 20°C; (d) Method 4: high humidity curing (relative humidity of 95%) at 25°C. For control mixes of CEM-I (ordinary cement) only water-curing and constant moisture curing were used corresponding to common practice. The cube compressive strength (100 mm^3^ cubes), tensile splitting strength and water absorption (30 min of immersion) were determined using BSI (2009b, 2009c, 2011), respectively.

**Table 1.** Mix design (kg m^–3^).

Mix #	Mix ID	CEM-I (kg)	GGBS (kg)	PSA (kg)	River sand (kg)	Coarse aggregate (<10 mm) (kg)	Water glass Na_2_SiO_3_ solution (kg)	KOH solution (kg)	Added water (kg)	w/b ratio	l/s ratio^a^	Slump (mm)
1	WG_71_KOH_46	0	415	0	784	1039	71	46(1M)	137	0.33	0.49	230
2	WG_71_KOH_32_P_16 (PSA in slurry form)	0	415	16	784	1039	71	32(1M)	137	0.33	0.5	N/A (collapse)
3	WG_71_PSA (sl)_16 (PSA in slurry form)	0	415	16	784	1039	71	0	202.4	0.49	0.58	N/A
4	WG_112_PSA (sl)_16 (PSA in slurry form)	0	415	16	784	1039	112	0	186.4	0.45	0.58	35 (195 after remixing)
5	WG_112	0	415	0	784	1039	112	0	202.4	0.49	0.58	N/A (collapse)
6	CEM-I	415	0	0	784	1039	0	0	224	0.54	0.54^b^	200
Complementary mixes (7-day tests only)												
7	WG_112_PSA (sl)_32 (PSA in slurry form)	0	415	32	784	1039	112	0	186.4	0.45	0.58	N/A (collapse)
8	WG_112_PSA(p)_16_l/s_0.43 (PSA in powder form)	0	415	16	784	1039	112	0	139	0.33	0.43	25
9	WG_112_l/s_0.43	0	415	0	784	1039	112	0	135	0.33	0.43	–

a l/s is the liquid/solid ratio adjusting the water/binder ratio (w/b) to include water and solids in activator solutions.

b in the AAC mixes l/s varied (0.5 and 0.58 respectively), so the average l/s ratio of 0.54 was used for the control CEM-I mix.

GGBS: ground granulated blastfurnace slag; PSA: paper sludge ash; WG: waterglass; AAC: alkali-activated cement.

## Results and discussion

### Cube compressive strengths

**Figure 1.**
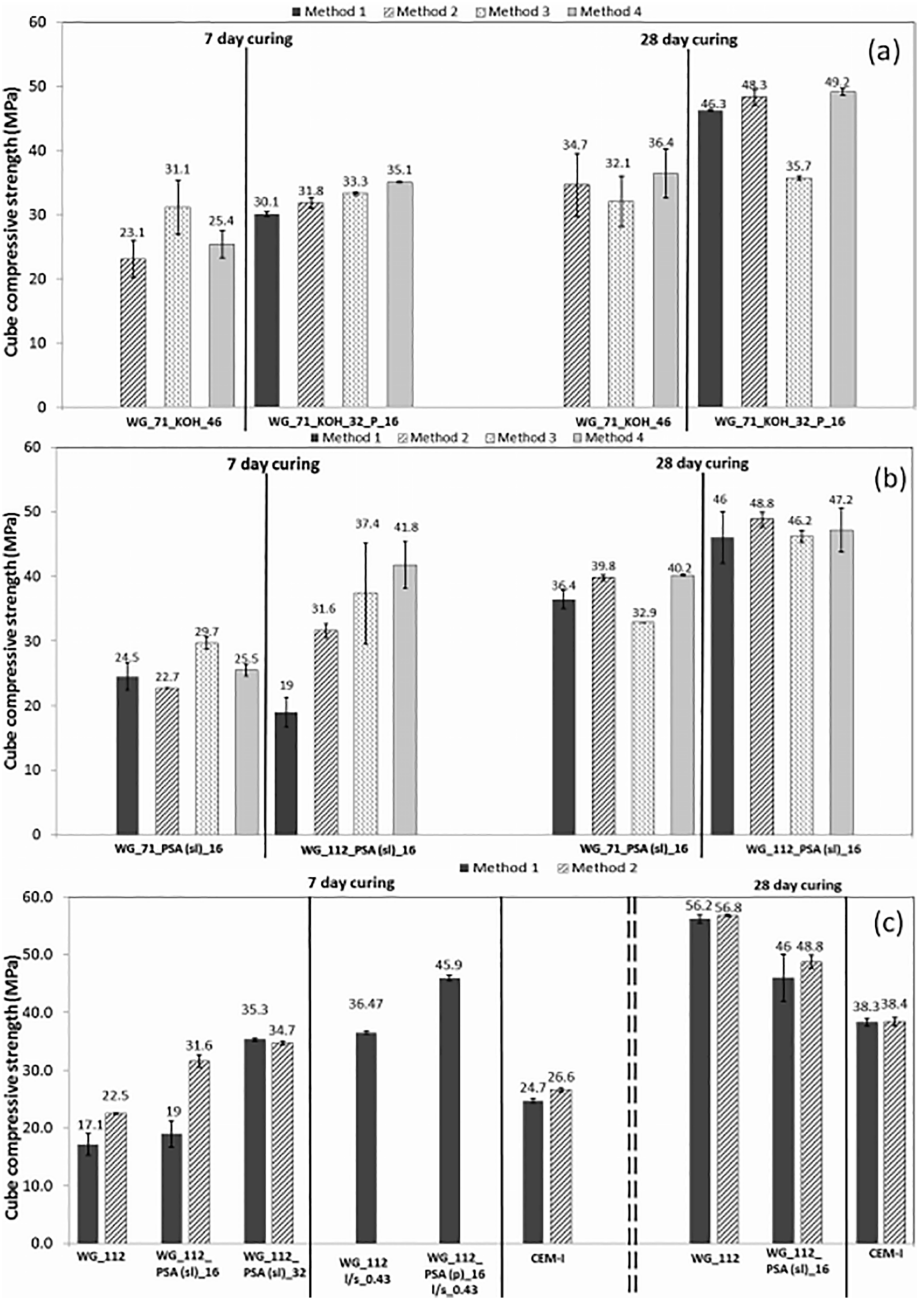
Cube compressive strength: (a) KOH mixes, (b) mixes with WG-effect of WG content, (c) effect of PSA on WG mixes, and control mix strengths. WG: waterglass; PSA: paper sludge ash.

From Figure 1 it can be seen that, comparing control mix 1 (WG and KOH only) with mix 2 where PSA partly replaced KOH, mix 2 had higher strengths at all ages and curing methods (except perhaps method 3 considering the error magnitude). It could thus be possible and beneficial in terms of strength to partly replace commercial KOH by waste PSA with possible cost savings. Moreover, KOH mixes set fast and had to be cast into moulds promptly. Rapid setting time of alkali-activated slag cement mixes and a quick loss of slump if the mix is not in motion is a known issue (Wang et al., 1994). The use of Ca(OH)_2_ was suggested to lengthen the setting time of alkali-activated slag cement mixes (Douglas et al., 1991); this was observed for KOH mixes with PSA. The benefits in terms of strengths in mixes with WG and PSA only (mixes 3, 4, 7, 8) are less clear: mixes with PSA had higher early strengths than the respective AAC mixes without PSA. However, at 28 days all WG mixes without PSA acquired higher strengths, implying that PSA hindered later strength gain. This is unlike observations for soil stabilisation, where cement mixes with WG+PSA consistently outperformed WG mixes of the same WG content (Mavroulidou et al., 2019, 2020). A detailed mineralogical, chemical and microstructural analysis is required to explain this. Early strength development and higher strengths than ordinary cement mixes due to the presence of metakaolin and portlandite were also observed when PSA was used as a supplementary cementitious material in Portland cement mixes (Kadu and Gajghate, 2016; Mavroulidou and Awoliyi, 2018; Yousuf et al., 2014). Early strength gains can be of practical importance for precast concrete and for rapid construction projects; for these PSA could be advantageous as strengths at all ages are adequate for structural concrete, and higher than the indicative ordinary cement CEM-I mix, even if the l/s ratio of the PSA mixes was slightly higher. By increasing the WG content for the same amount of PSA higher strengths were developed (Figure 1b) due to the increased dissolution rate of solid aluminosilicate with increased alkali content. Some small strength gains were also noted when increasing the PSA content (Figure 1c): in future work further PSA contents will thus be tested (in the first stage PSA was kept low anticipating workability issues). The effect of curing method was variable; for early strengths the two best methods were 4 (high humidity) or 3 (temperature) – for lower WG content-. At later times, method 3 led to limited further strength development (for most mixes, >90% of the 28-day strength developed in the first 7 days of curing except for the PSA mix with higher WG content); this agrees with Bakharev et al. (1999) and Gu et al. (2014) on temperature curing of alkali-activated slag concrete. Generally, method 1 gave the lowest early strengths; samples were still soft after 24 h curing, and some were damaged when placed in the water bath. In later work (e.g. complementary mixes) samples were thus left in the moulds for 72 hours at a constant moisture, then put in water. For the 28-day strength (the nominal strength used for concrete design) the best curing methods were 4 or 2 (constant humidity); due to water availability for hydration reactions, water method 1 had the highest strength development in time despite possible alkali ion leaching into the water.

### Tensile strength and water absorption

**Figure 2.**
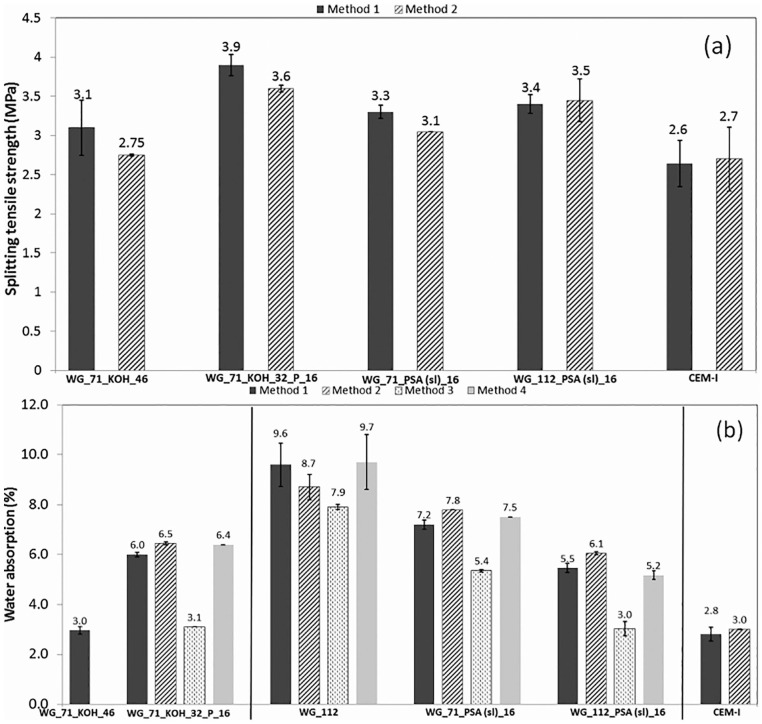
Indicative 28-day tensile strengths (a), and water absorption (b). WG: waterglass; PSA: paper sludge ash.

**Figure 3.**
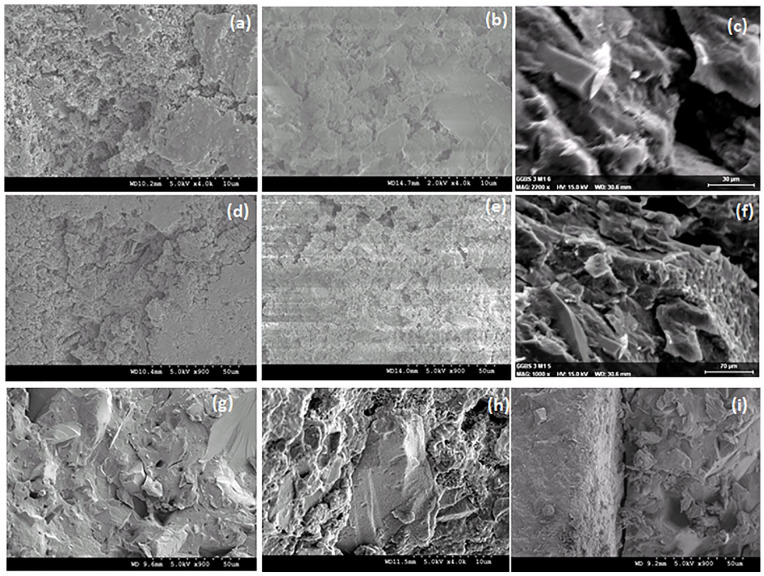
Indicative scanning electron microscopy (SEM) photos: (a, d) control mix CEM-I (method 1); (b, e) mix 1 (method 1); (c, f) mix 2, method 1; (g) mix 1, method 2; (h) mix 1, method 3; and (i) mix 2, method 2.

Figure 2(a) shows indicative splitting tensile strength (f_sp_) results; AAC mixes had f_sp_ values of *c*. 7–9% of the compressive strength (*c*. 7% for CEM-I); in most cases method 1 had higher strengths. Generally, AAC mixes showed a higher water absorption than the control CEM-I mix (see Figure 2(b)), except Mix 2 (KOH+WG) due to the similar structure to that of the control mix (i.e. fairly uniformly distributed hydration products – see scanning electron microscopy (SEM), Figure 3 (a) vs. (b) and (d) vs. (e)). Method 3 mixes showed the lowest water absorption, possibly due to reduced drying shrinkage, according to Bakharev et al. (1999). Mixes with PSA had improved (lower) water absorption compared to mix with WG only (including lower absorption by capillary rise, and generally also lower porosity –helium porosimeter tests – not shown here for brevity); thus, there is an advantage of using PSA in the WG mixes. Based on Le Châtelier test (BSI, 2016), there were no soundness issues due to CaO in PSA.

### Scanning electron microscopy

SEM at 28-day curing confirms the development of hydration products for AAC mixes, justifying their good strength: mix 1 has a dense matrix of hydration products (Figure 3(b), (e), (g)), similar to CEM-I control mixes (Figure 3(a), (d)), which had a dense honeycombed structure due to hydration product formation; in mix 2 large portlandite crystals are seen (WG+PSA) in addition to hydration gel network of CSH/C(A)SH type (Figure 3(c), (f)). Microcracks and dissolution/other interconnected voids are noted in the AAC mixes (see Figure 3 (c) and (f) to (i)), as opposed to the CEM-I control mixes (Figure 3(a), (d)); these may contribute to the increased water absorption of the AAC mixes compared to the CEM-I control mixes. However, voids and microcracks appear to be bridged by/filled with crystals or hydration gel networks (see Figure 3(f), (h), (i)), which can justify the good strength of the AAC mixes despite the observed voids/cracks.

## Conclusion

This preliminary study assessed the feasibility of using PSA in activator systems of GGBS for structural concrete AAC. Curing at high available moisture and ambient temperature was successful for all AAC mixes, and overall good strengths were obtained. In WG activator mixes, the advantage of PSA was the higher early strengths and lower water absorption than mixes with WG only. However, 28-day strengths were lower than mixes with WG only. Introducing PSA as partial replacement of KOH improved both 7 and 28-day strengths. Further mix optimisation and mechanical and durability testing accompanied by material characterisation is required to establish the advantages of using this waste material in AAC concrete.
